# Supplementary figures and images for: Pancreatic tumors exhibit myeloid-driven amino acid stress and upregulate arginine biosynthesis
Source: eLife. 2023 May 31;12:e81289. doi: 10.7554/eLife.81289 (PMC10260022; doi:10.7554/eLife.81289)

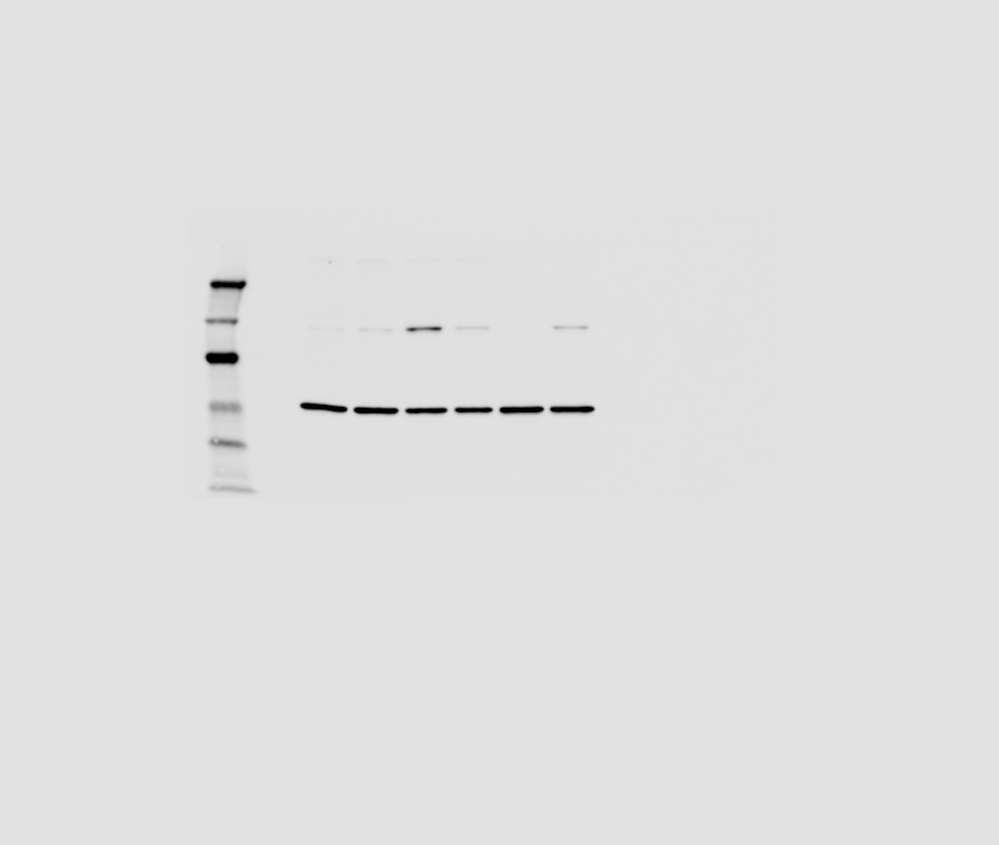

Supplement: Figure 1—source data 4. — Raw image files also included. [file elife-81289-fig1-data4.zip › Fig. 1 Source Data 4/Fig. 1 Source Data 4 Actin.tif]

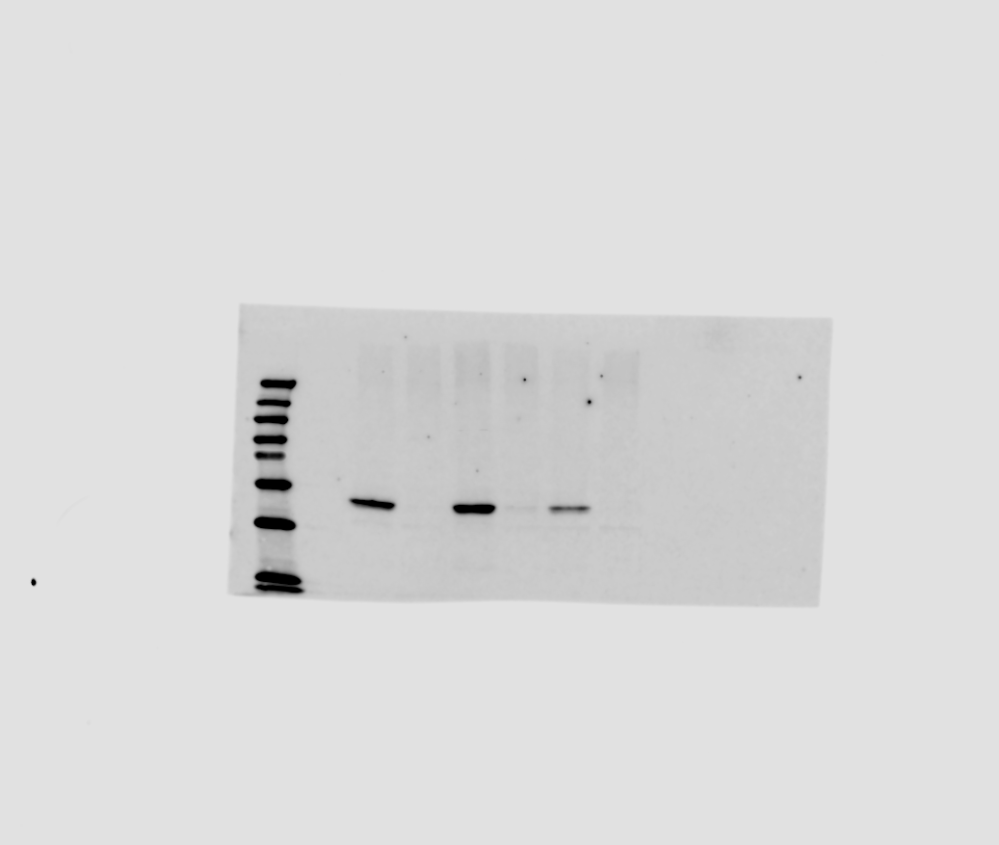

Supplement: Figure 1—source data 4. — Raw image files also included. [file elife-81289-fig1-data4.zip › Fig. 1 Source Data 4/Fig. 1 Source Data 4 ASS1.tif]

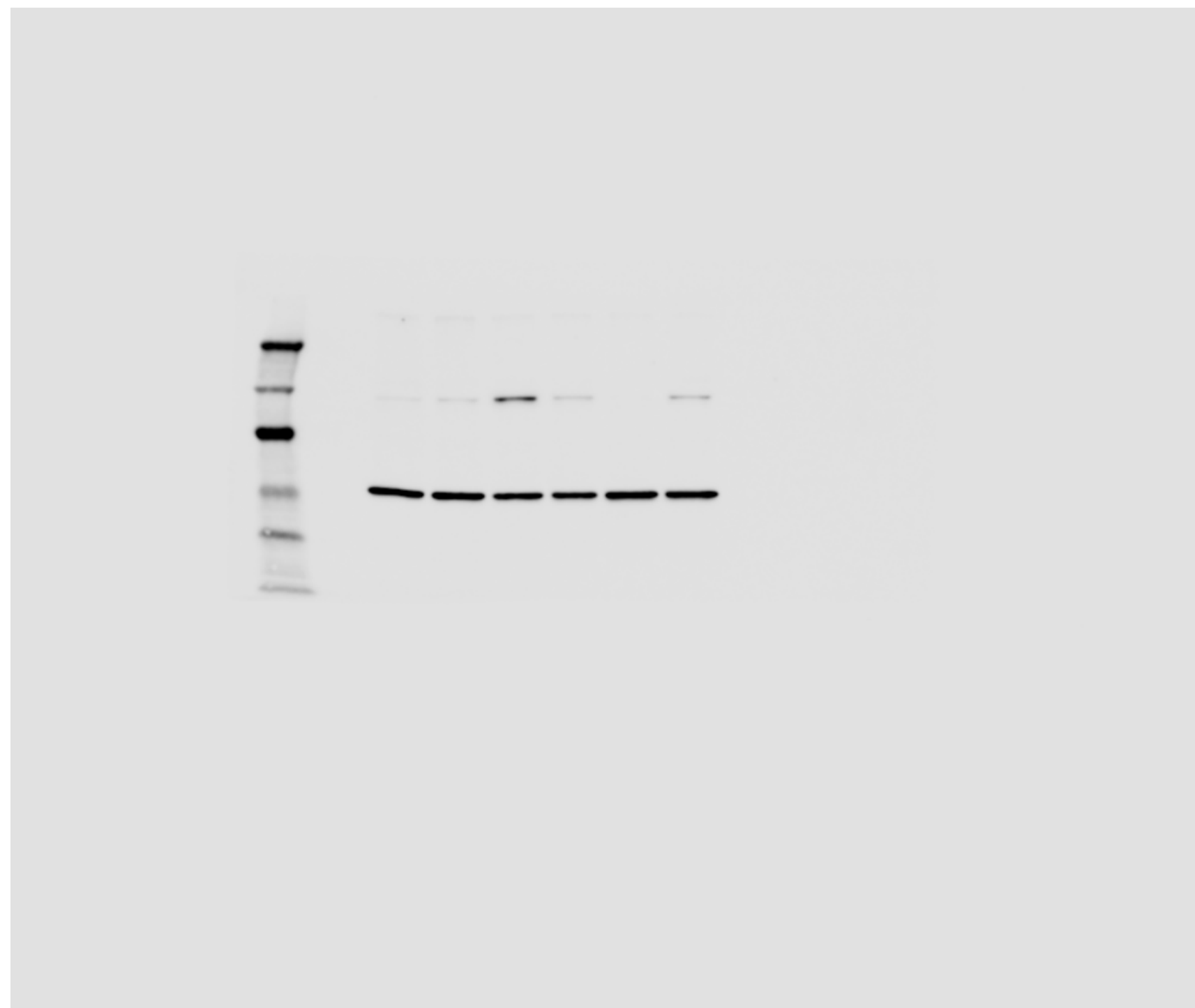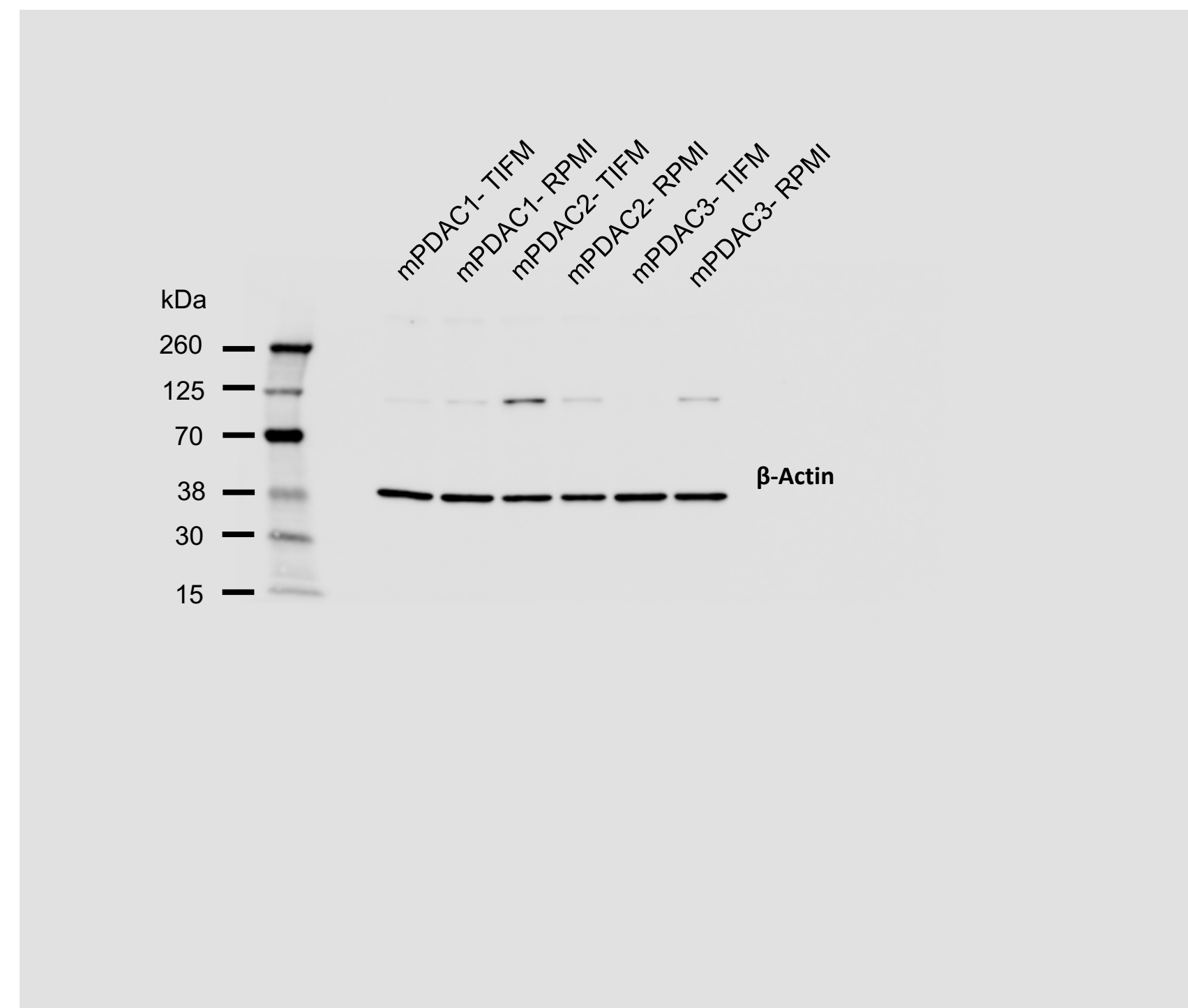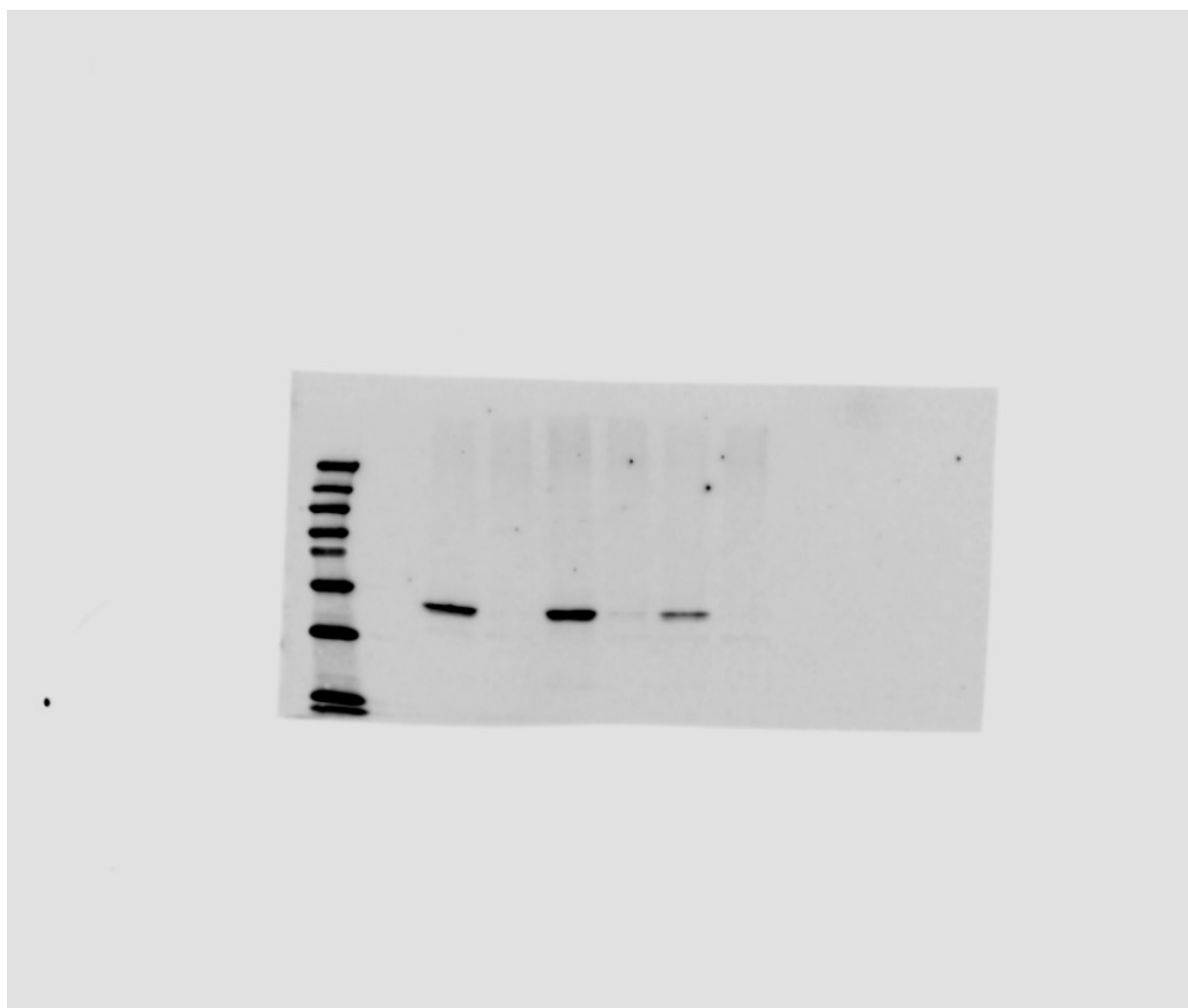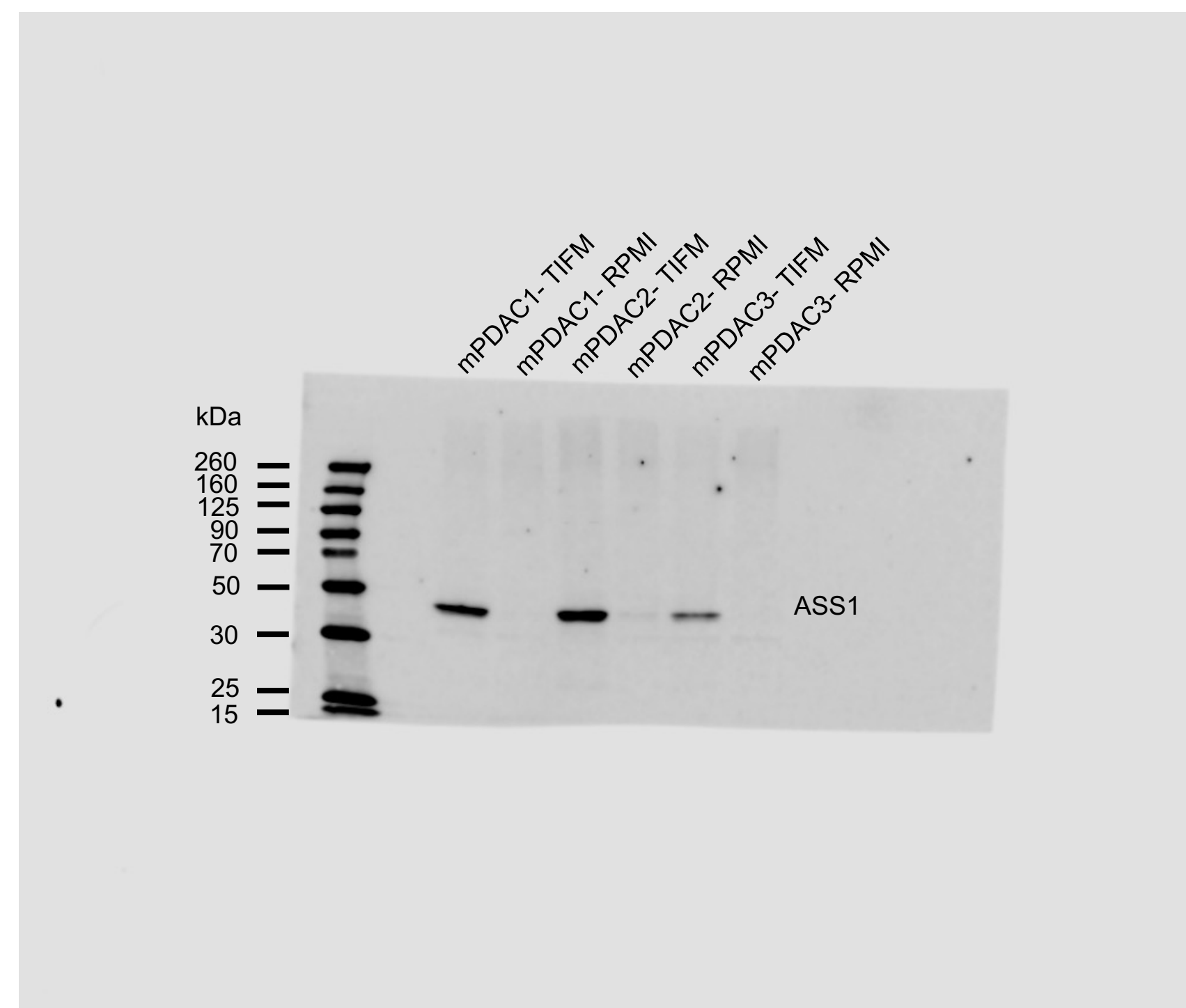

Supplement: Figure 1—source data 4. — Raw image files also included. [file elife-81289-fig1-data4.zip › Fig. 1 Source Data 4/Fig. 1 Source Data 4.pdf]

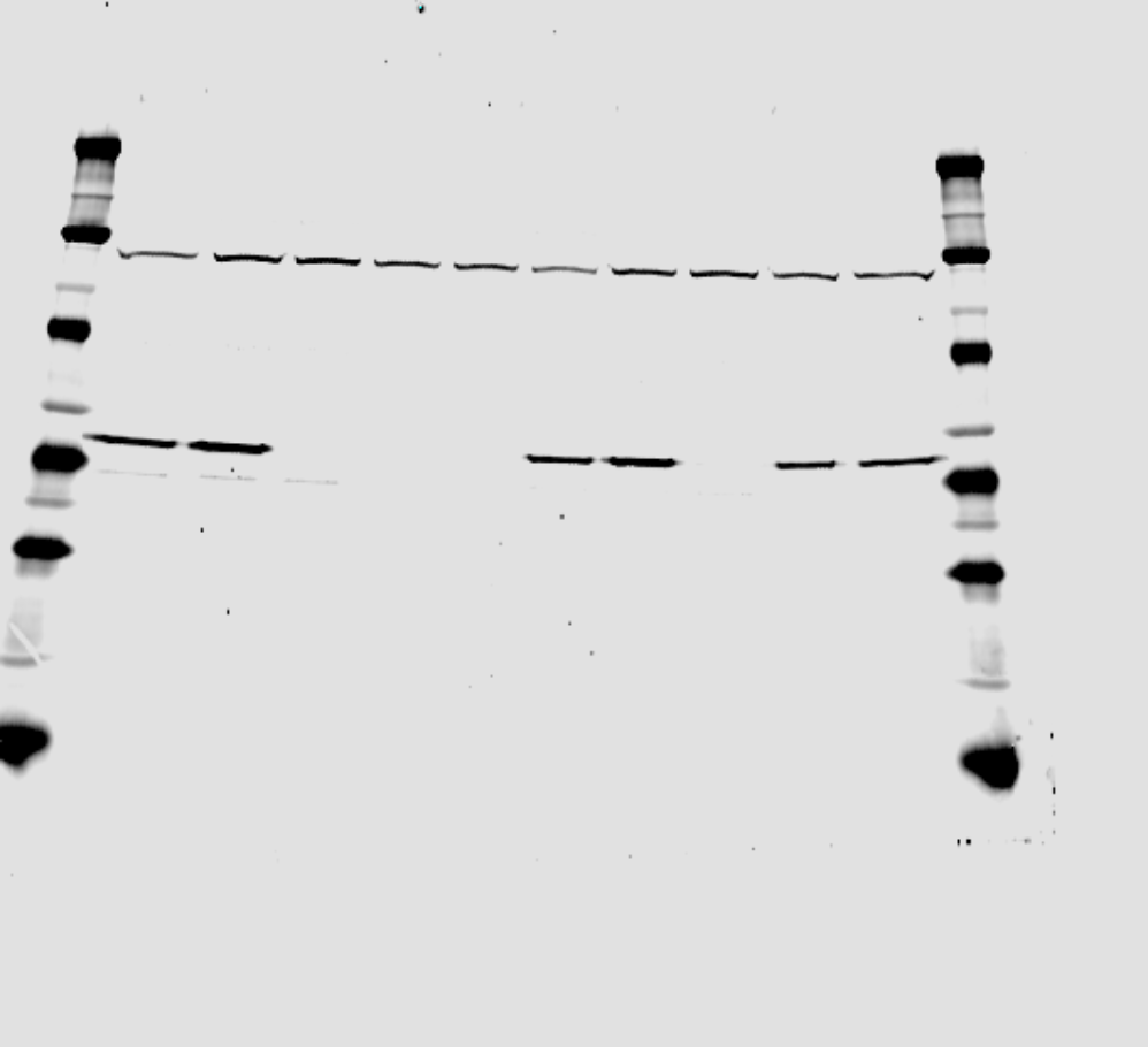

Supplement: Figure 2—source data 4. — Immunoblot analysis was performed in cells after 24 hr of growth in Tumor Interstitial Fluid Medium (TIFM) or TIFM + 100 μM as indicated. Raw image files also included. [file elife-81289-fig2-data4.zip › Fig. 2 Source Data 4/Fig2. Source Data 4.tif]

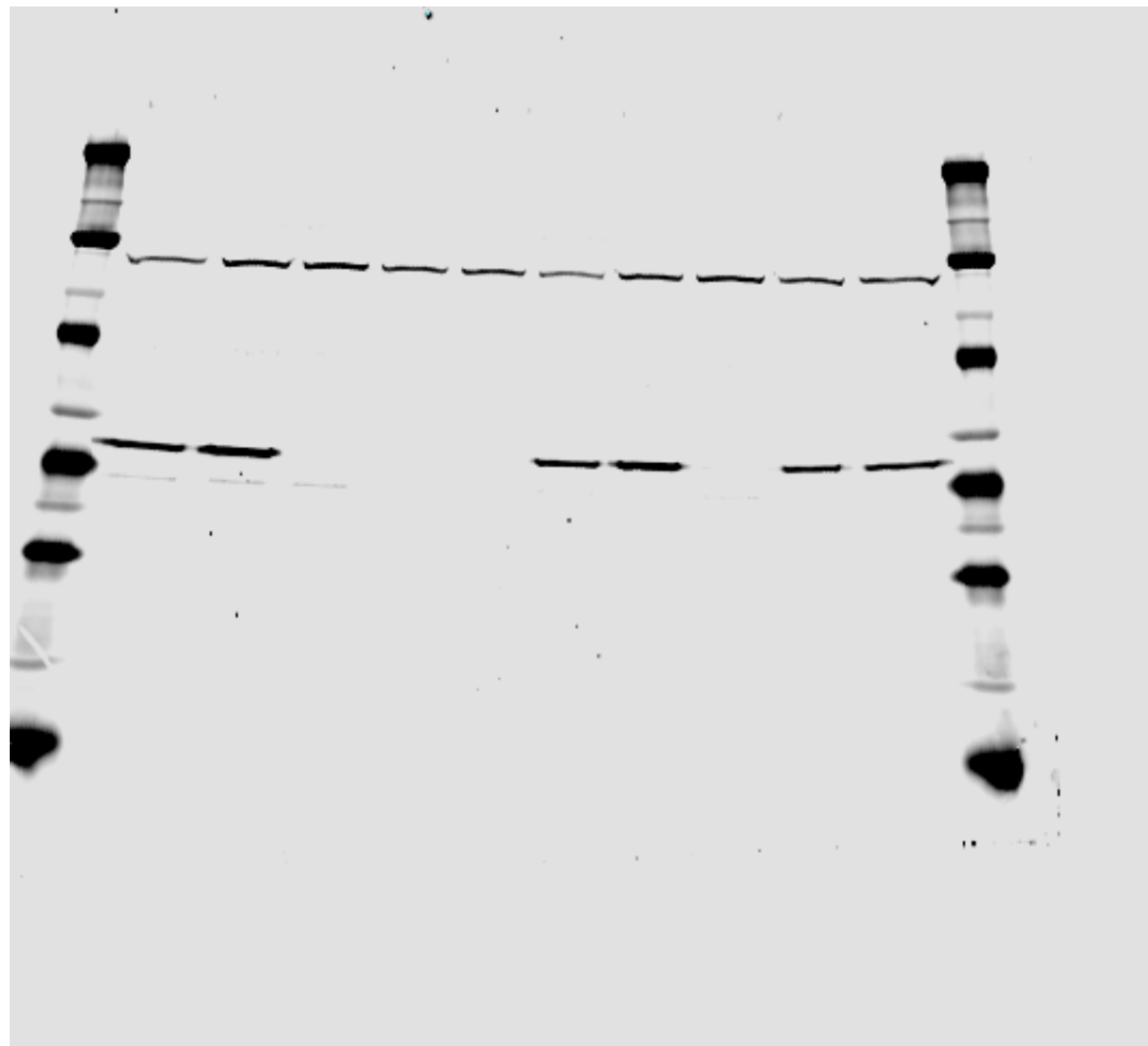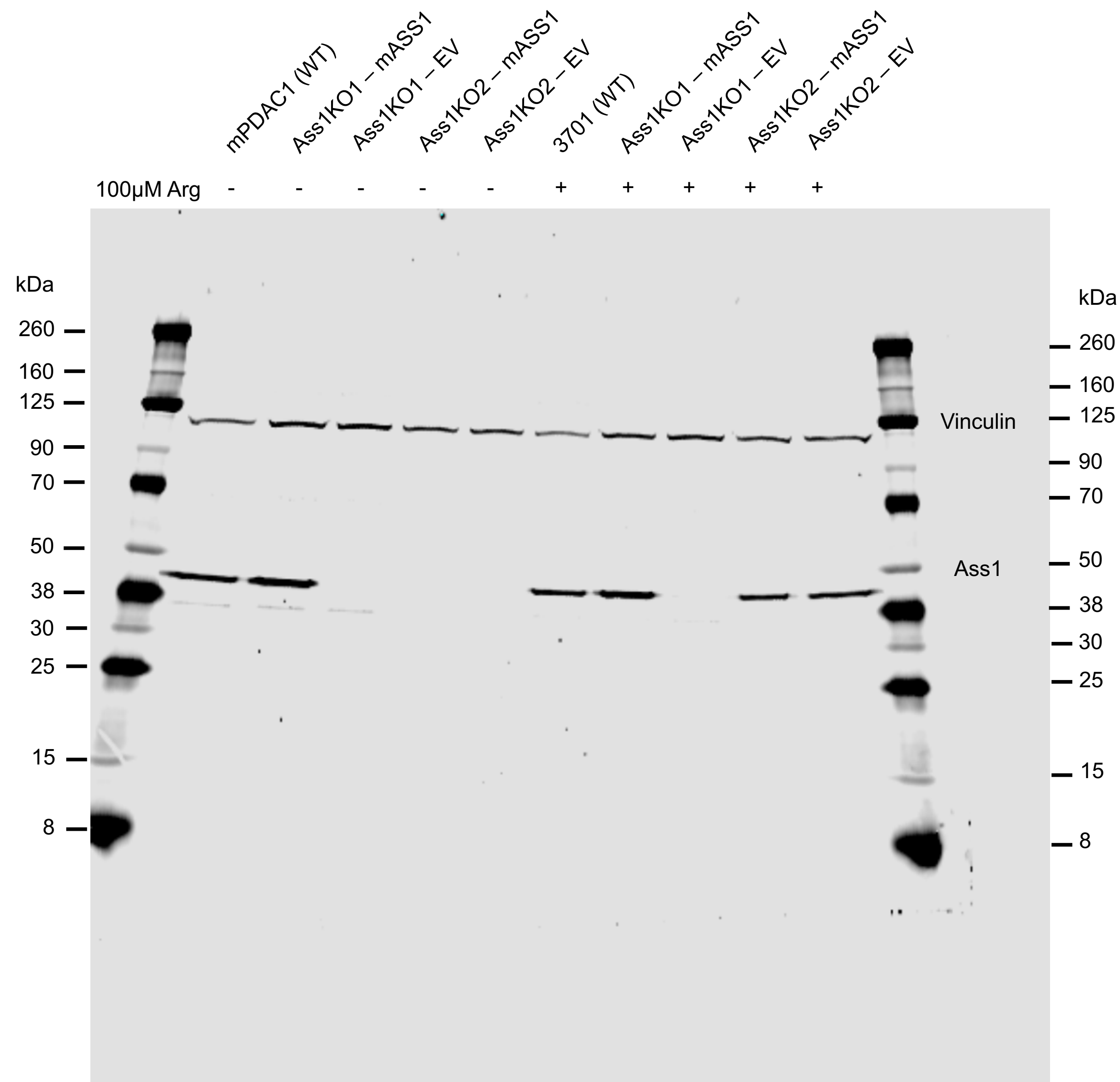

Supplement: Figure 2—source data 4. — Immunoblot analysis was performed in cells after 24 hr of growth in Tumor Interstitial Fluid Medium (TIFM) or TIFM + 100 μM as indicated. Raw image files also included. [file elife-81289-fig2-data4.zip › Fig. 2 Source Data 4/Fig2. Source Data 4.pdf]
